# Supplementary material for: Public involvement and co-design of longitudinal studies of sleep health alongside young people with rare genetic conditions
Source: Res Involv Engagem. 2026 Jul 13;12:113. doi: 10.1186/s40900-026-00935-z (PMC13359642; doi:10.1186/s40900-026-00935-z)
Supplement: Supplementary file 1 — Supplementary Material 1: Additional file 1 (pdf): PPI workshops information pack. This contains the information co-produced by the Lived Experience Advisory Panel to explain to children and young people what to expect at the hands-on PPI workshops, who they would meet, what activities they would do, a sensory map of the venue and a sticker chart to mark their progress through the workshop. Accessible at Open Science Framework: https://osf.io/hkprx/files/osfstorage/69cd301626c3d97ee4745504 [file 40900_2026_935_MOESM1_ESM.pdf]

SLEEP DETECTIVES

CAN YOU HELP DETECTIVE OTTERLEE SLEEPEE AND HER TEAM OF RESEARCHERS?

WE ARE SEEKING A SQUAD OF SNOOZE SLEUTHS TO CRACK THE CASE OF "THE ELUSIVE EQUIPMENT"!

WORKSHOP: 8-9TH JUNE

CAN YOU TRY
OUR EQUIPMENT AND
GAMES
AND TELL US
WHAT YOU THINK?

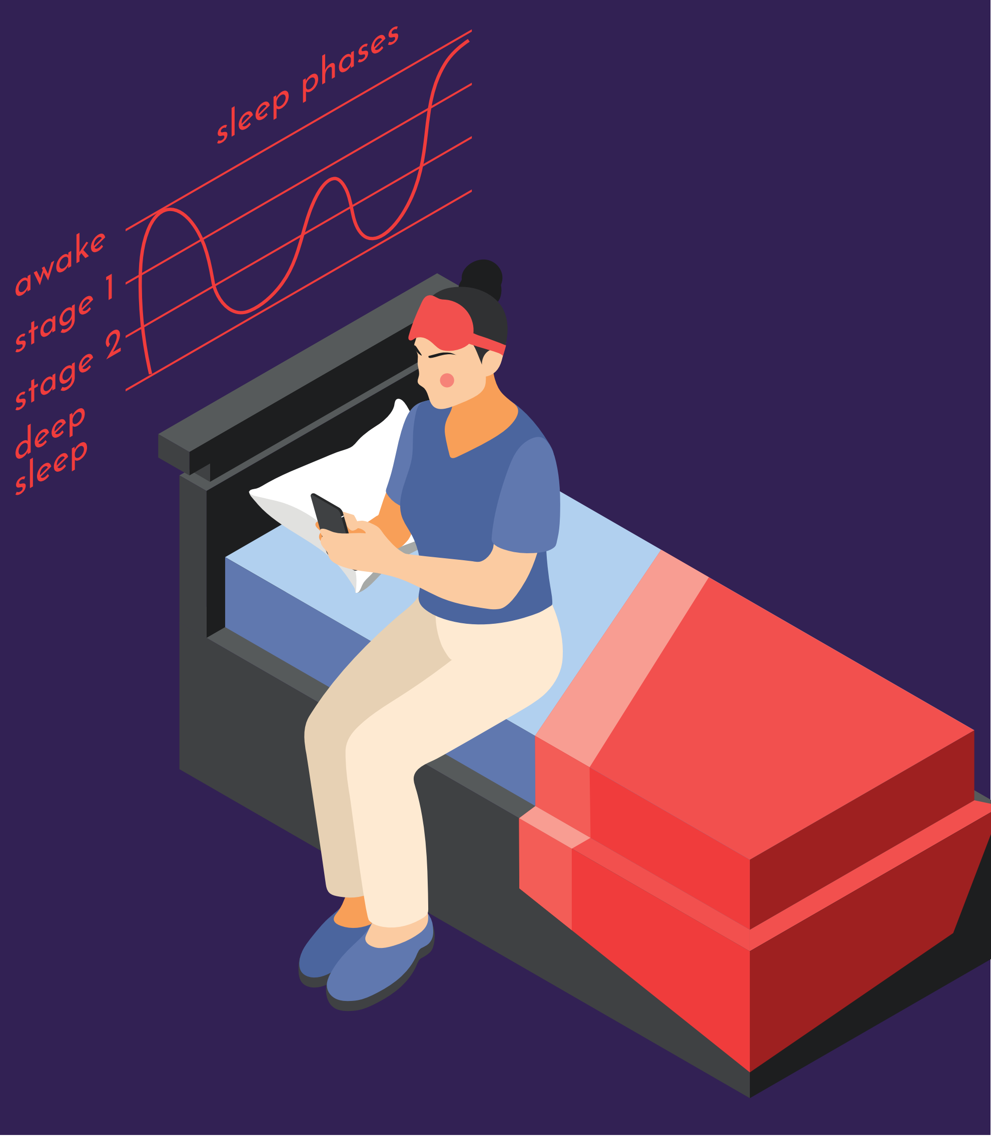

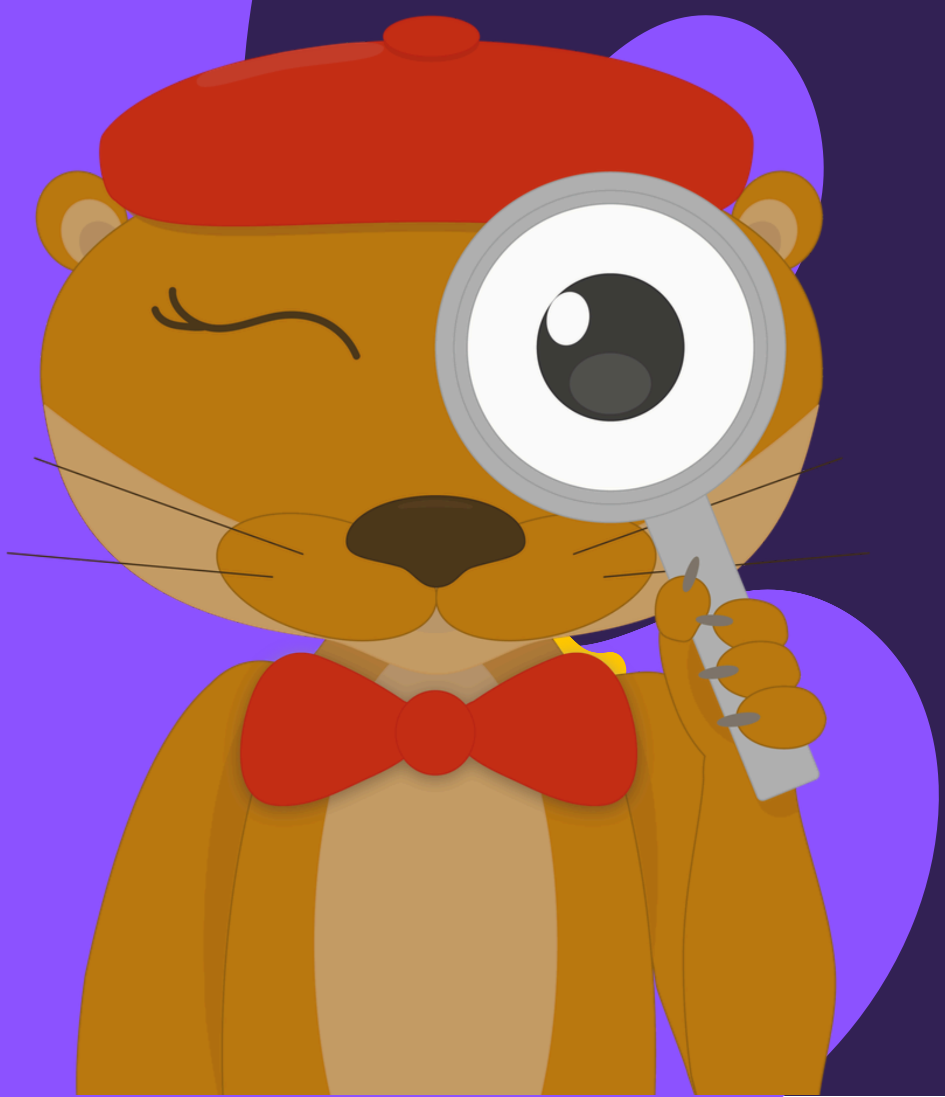

Bring your families:
CANYNGE HALL
Bristol

Sleep Detectives

Unravelling the secrets of sleep with Detective Otterlee Sleepee

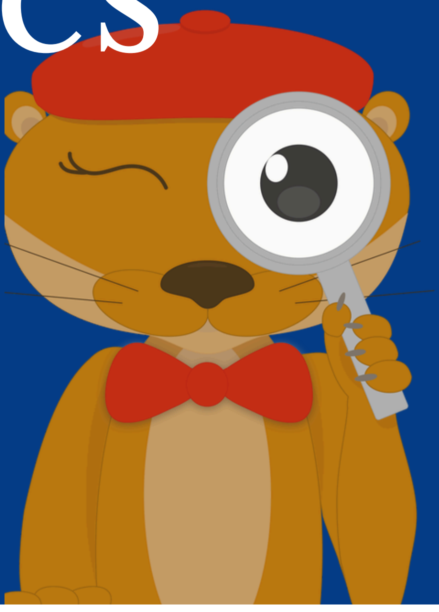

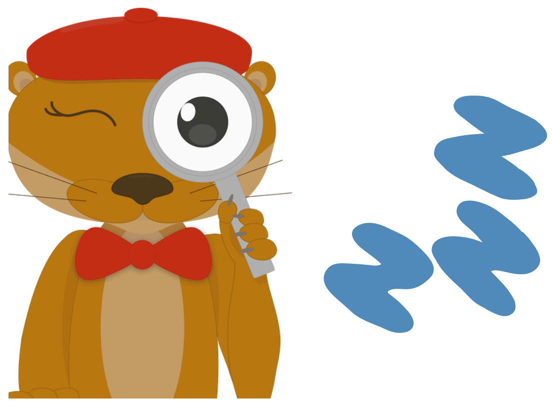

Detective Otterlee Sleepee, and her group of researchers are interested in how different children might sleep differently.

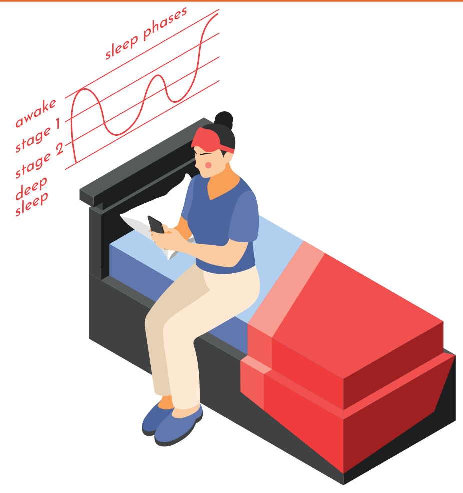

To understand more about how children sleep, researchers need to use special equipment.

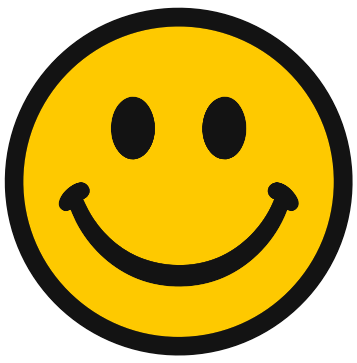

The researchers need to make sure that the equipment they use is the best, and that it is comfy.

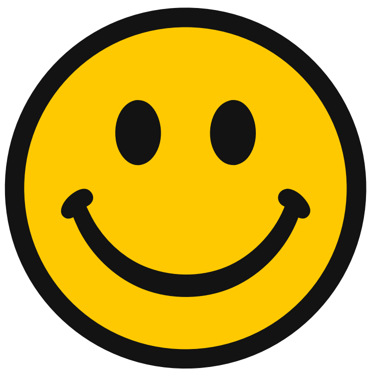

Can you be a sleep detective, and help Detective Otterlee Sleepee and her gang of researchers, by trying out their equipment?

University of BRISTOL

CARDIFF UNIVERSITY

What will happen on the weekend?

Friday, Saturday and Sunday

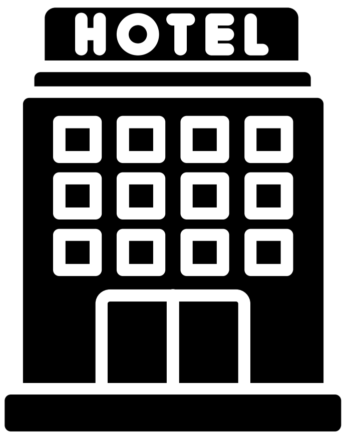

You will travel to Bristol with your families and stay in a hotel.

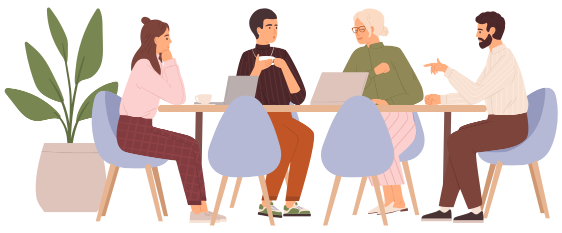

You will come to our sleep space where you will meet some other families who are also being sleep detectives for the weekend.

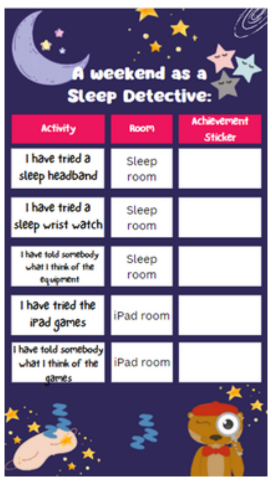

A weekend as a Sleep Detective:

Activity	Room	Achievement Sticker
I have tried a sleep headband	Sleep room	
I have tried a sleep wrist watch	Sleep room	
I have told somebody what I think of the equipment	Sleep room	
I have tried the iPad games	iPad room	
I have told somebody what I think of the games	iPad room	

The researchers will ask you to try some equipment and games. You will get stickers for completing these activities.

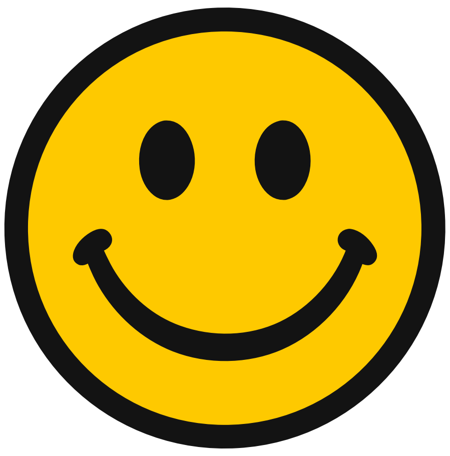

You might try some sleep headbands on, or try a sleep wristwatch. The researcher might ask you to put a headband on and lie back so you can tell if it is comfy.

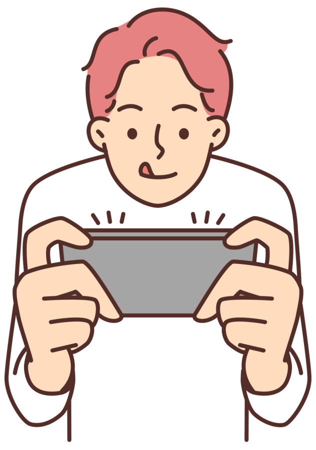

You will also try some games on iPads. The researchers will show you what to do and ask you if you liked playing the games.

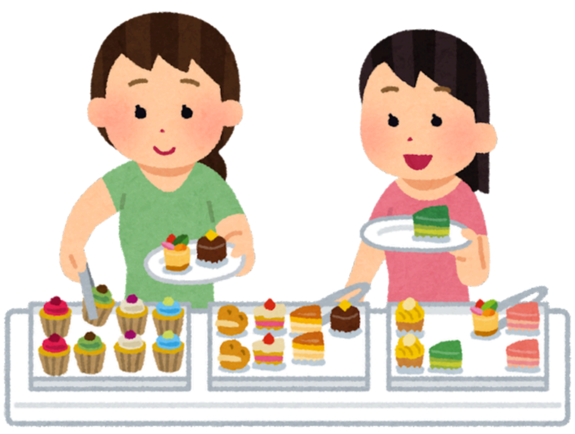

We will have lunch at 1pm - you can help yourselves to what you want to eat from our buffet.

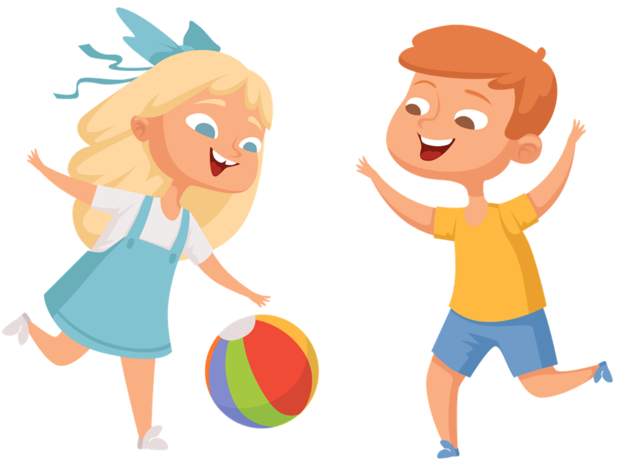

You can also play jigsaws, ball games, lego and colouring. Or you can chill out in our refreshment room or sensory space.

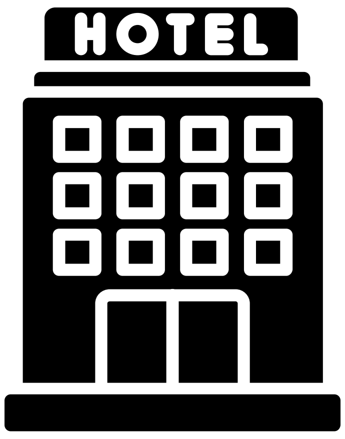

You will stay in your hotel again.

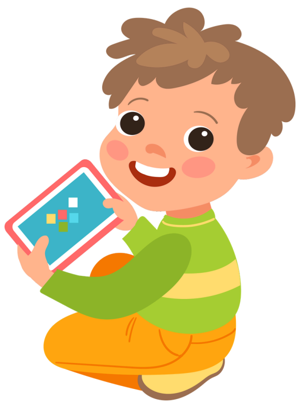

You will come back to the sleep space in the morning to do any activities you didn’t have time to do the day before.

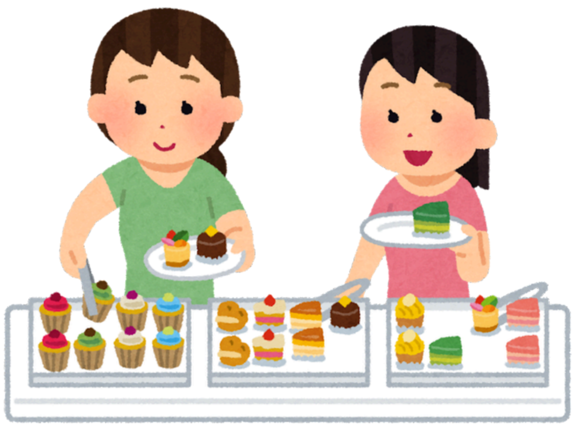

We will have lunch at 12pm - you can help yourselves to what you want to eat from our buffet.

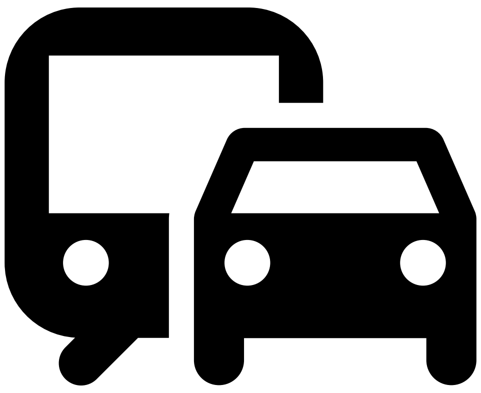

You will go home with your families. Some of you will come in the car, some of you will go on a train.

Sleep Detectives

Workshop Activity List

Please take part in any of the activities you like, but remember to help our researchers, by being sleep detectives. Try to complete all the activities on your sticker chart.

**Activity**	TRY OUT: Sleep headbands and sleep wristbands	TRY OUT: iPad Games	Design a character: drawing and colouring	Indoor ball play	Quiet sensory rooms: cushions, soft toys, books, calming music	Hands on quiet games: jigsaws, lego	Outdoor play: bean bag throw, coloured chalks, skittles, bubbles	Lunch 1-2pm	Private 1:1 chats
**Pictures**	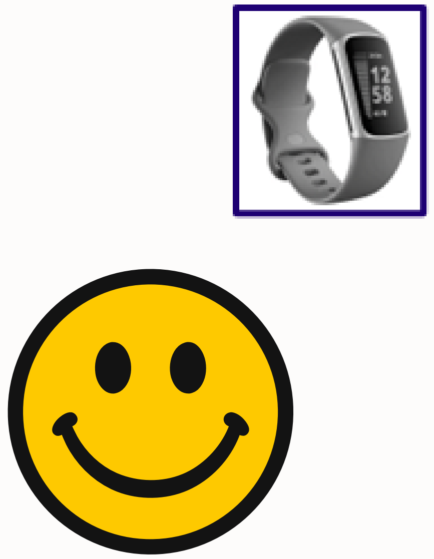	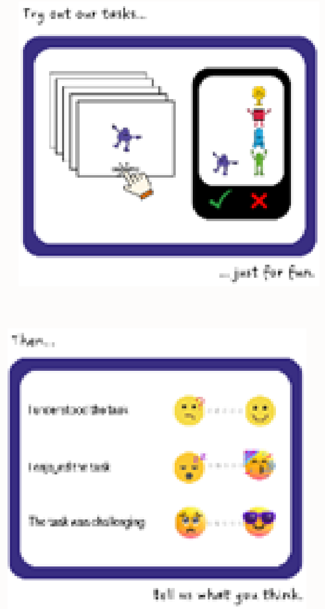	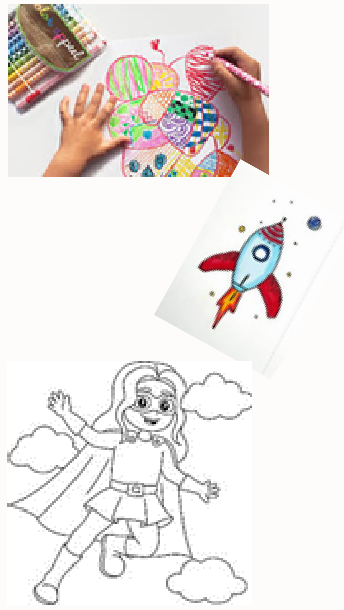	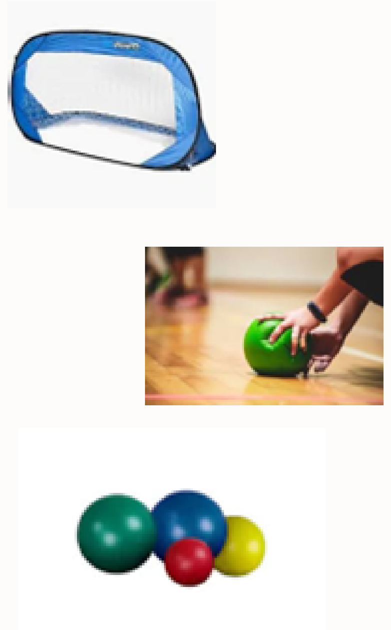	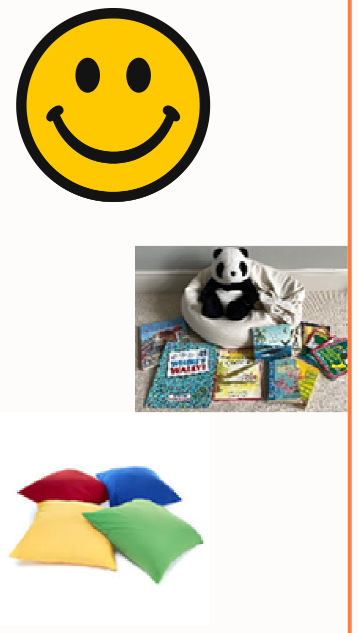	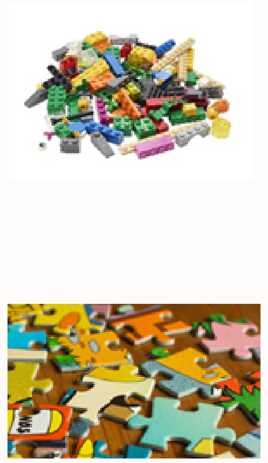	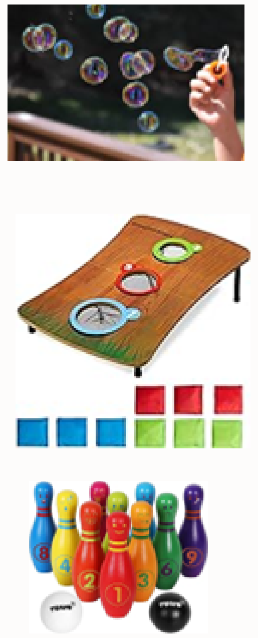	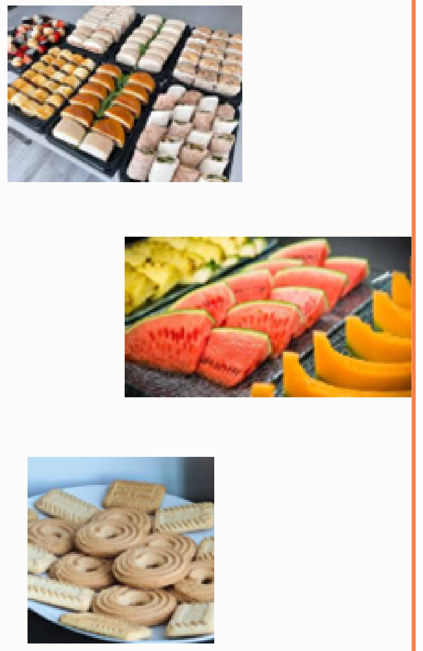	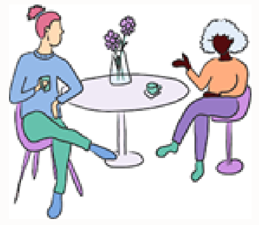
**Room**	Sleep room Lower ground	iPad room Lower ground	Refreshments Area - Lower Ground	Indoor ball room Lower Ground	Sensory room Ground floor	Hands on play room Ground Floor	Outside Yard Lower Ground	Tea Room and Outside Deck Ground Floor	G15 Ground Floor OR LG.23 Lower Ground

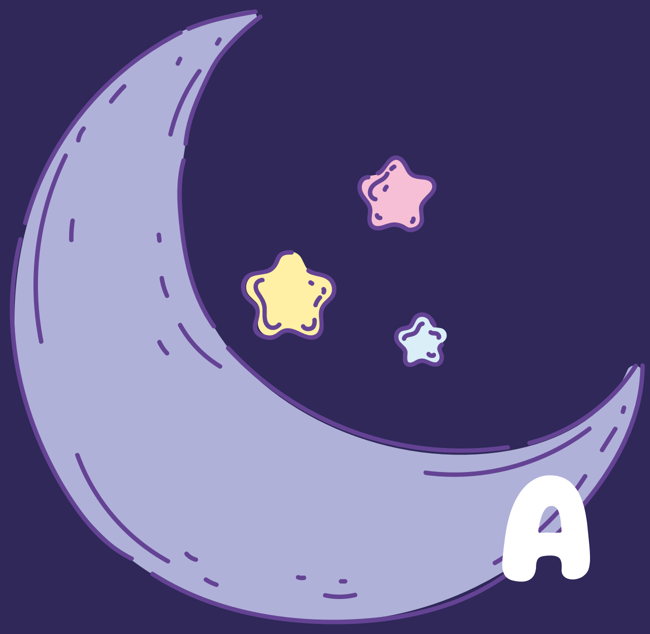

A weekend as a Sleep Detective:

Activity	Room	Achievement Sticker
I have tried a sleep headband	Sleep room	
I have tried a sleep wrist watch	Sleep room	
I have told somebody what I think of the equipment	Sleep room	
I have tried the iPad games	iPad room	
I have told somebody what I think of the games	iPad room	

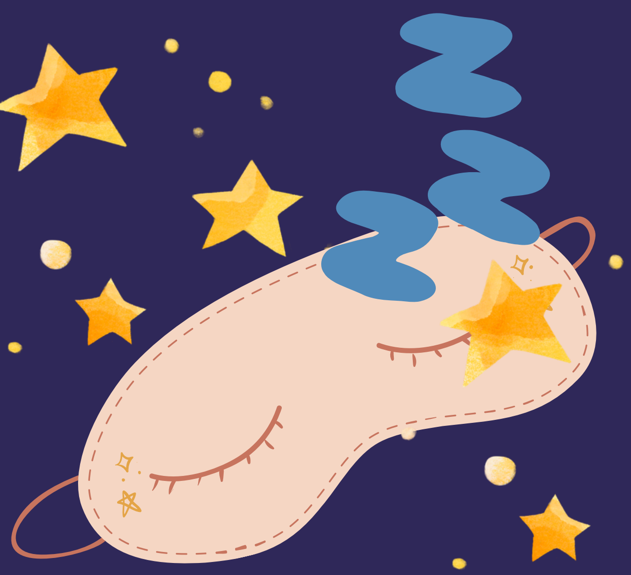

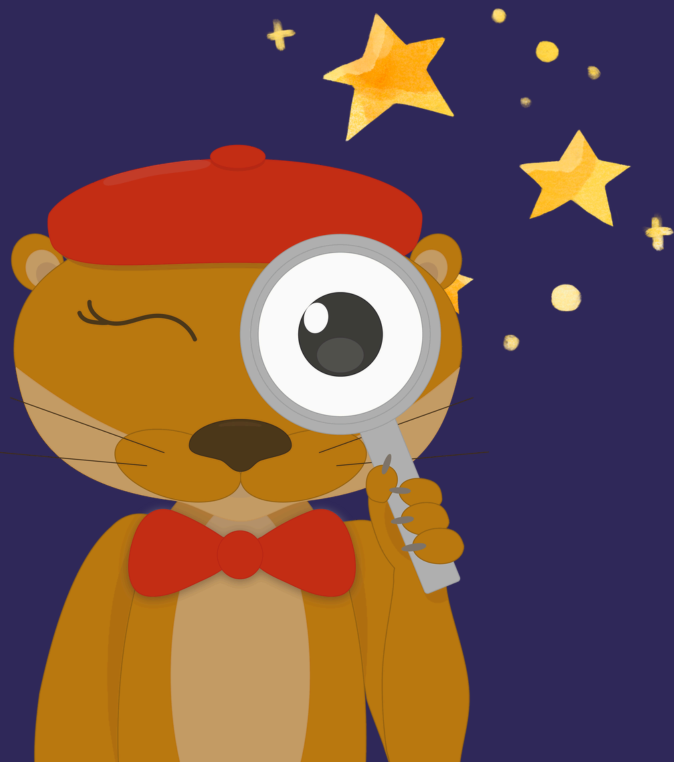

Canynge Hall - Bristol

Here is a floor plan of the building where our sleep detectives workshop will take place. This is a sensory map so you can see which rooms might be noisy, or have bright lights, and so you can see the quiet spaces to relax.

Ground Floor

1. You will enter onto this floor of the building.
2. NAME and NAME will be on reception (see their pictures)
3. Rooms G11 and G12 will be quiet spaces where people can relax
4. Room G12 will be a quiet multisensory space
5. Room G11 will be a quiet space where you can do activities like lego, and jigsaws
6. The tea room and refreshments area will be a quiet place where people can chat amongst themselves
7. Office G15 can be used as a quiet private space if you need to get out of the hustle and bustle.

Shhh! Quiet voices

Chatter

Natural Light

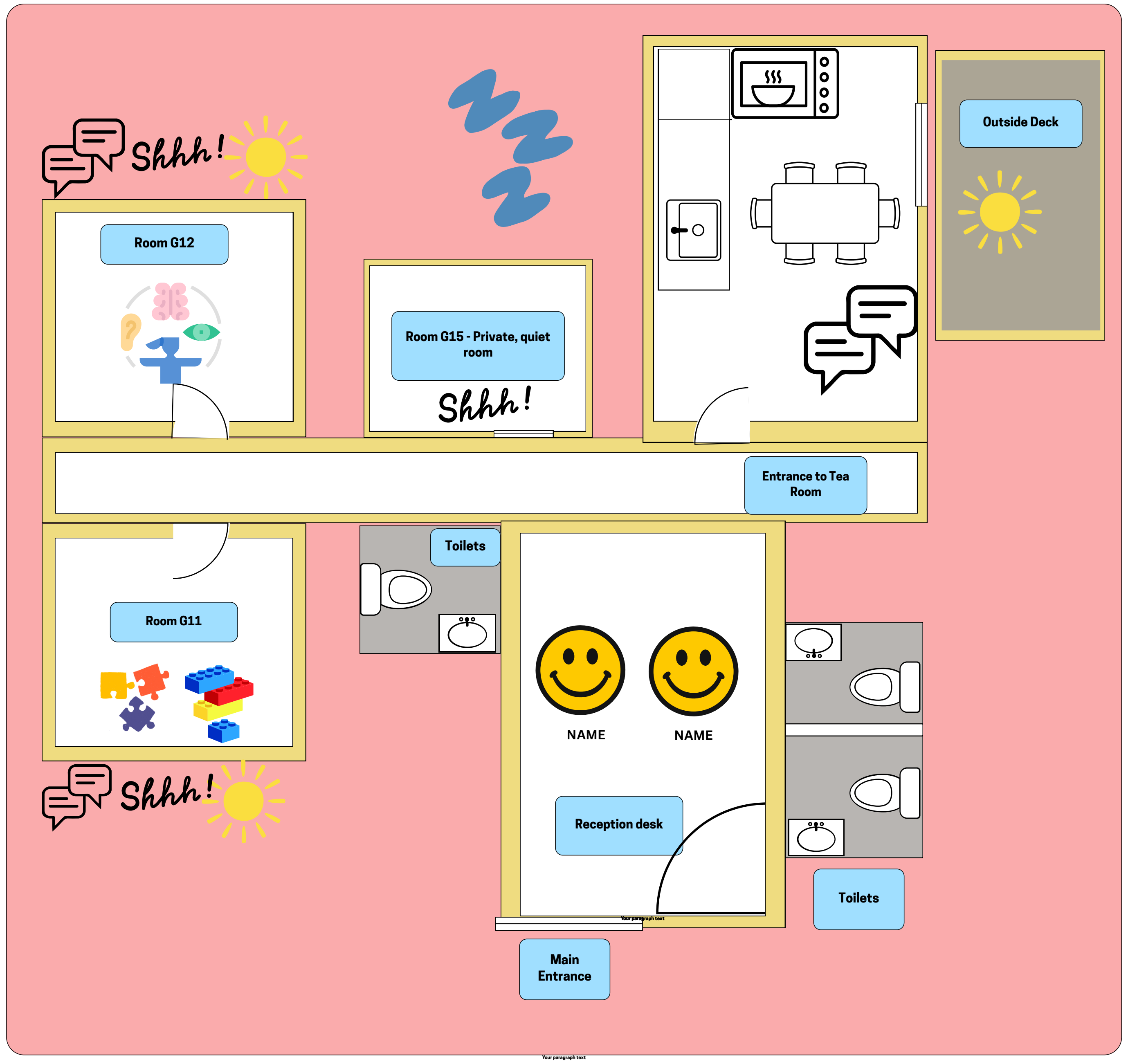

Lower Ground Floor

1. You will take stairs down to this level (there is a lift available)
2. Room LG.01 will be used for....
3. Room LG.03 will be our busy activity space - there will be some ipads to play on, and some arts and crafts activities
4. The outside yard will be a safe space for a run around and some ball games - it may be noiser here
5. Room LG08 A and B will be our detective rooms where you can try out our sleep equipment and games

Shhh! Quiet voices

Chatter

Noisy

Natural Light

Bright overhead lights

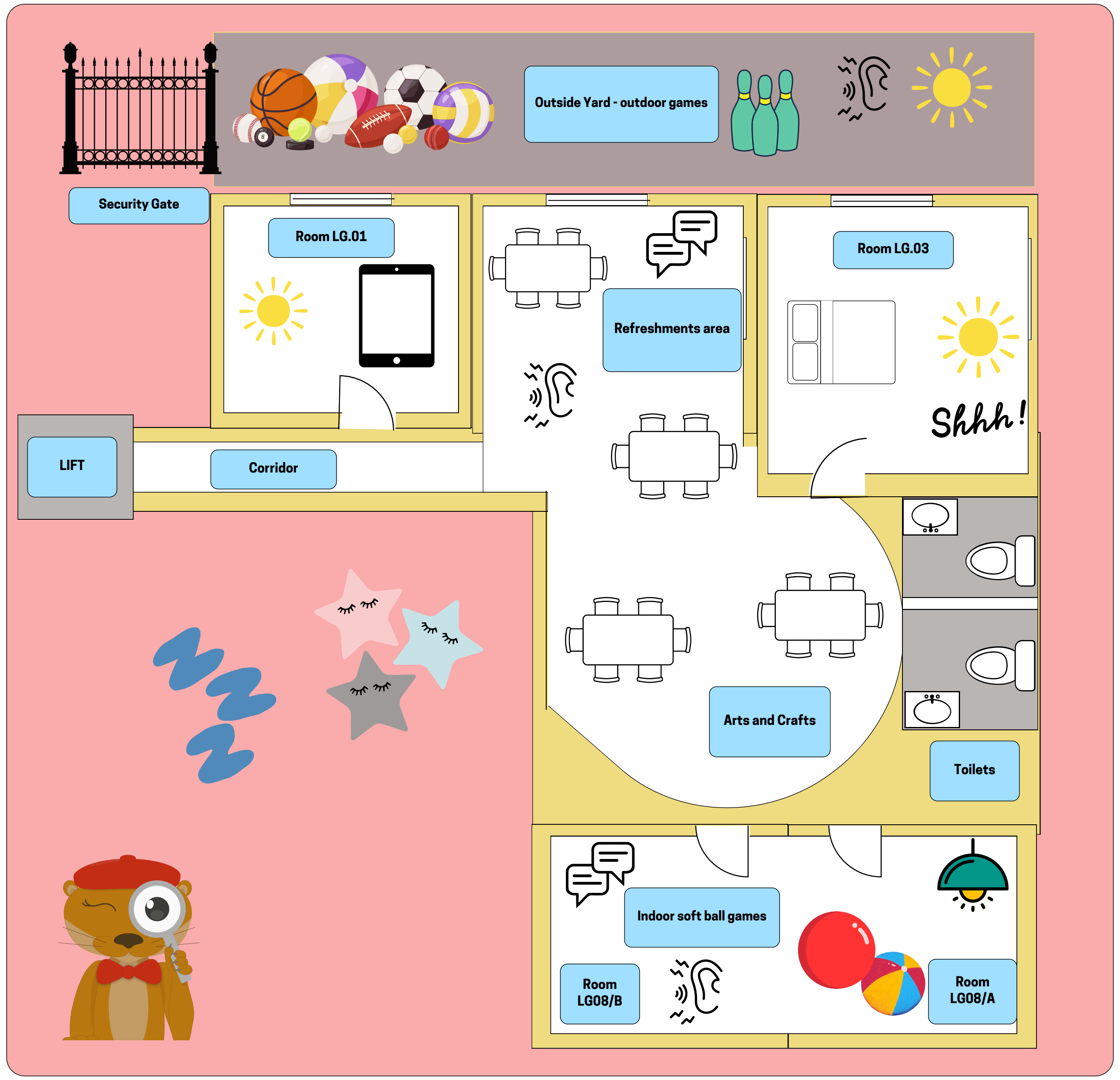

University of BRISTOL

SLEEP DETECTIVES

CARDIFF UNIVERSITY

Who might I meet?

Here are some of the team, who you might meet on the day!

Researchers

NAME NAME NAME NAME NAME

NAME NAME NAME NAME NAME

NAME NAME NAME NAME NAME

Charities

NAME

Canynge Hall Reception

NAME
